# *Achyrocline satureioides* Hydroalcoholic Extract as a Hypoallergenic Antimicrobial Substitute of Natural Origin for Commonly Used Preservatives in Cosmetic Emulsions

**DOI:** 10.3390/plants12102027

**Published:** 2023-05-18

**Authors:** Denisa Langová, Maria Angélica Mera Córdoba, Rodrigo Sorrechia, Julie Hoová, Zdeněk Svoboda, Renata Mikulíková, Marcos Antonio Correa, Rosemeire Cristina Linhari Rodrigues Pietro, Ivana Márová

**Affiliations:** 1 Institute of Food Science and Biotechnology, Faculty of Chemistry, Brno University of Technology, 612 00 Brno, Czech Republic; xcromanovska@fch.vut.cz (D.L.); hoova@fch.vut.cz (J.H.); xcsvoboda@vutbr.cz (Z.S.); mikulikova@fch.vut.cz (R.M.); 2 Department of Drugs and Medicines, School of Pharmaceutical Sciences, São Paulo State University UNESP, Araraquara 14801-902, São Paulo, Brazil; angelica.mera@unesp.br (M.A.M.C.); rodrigo.sorrechia@unesp.br (R.S.); marcos.a.correa@unesp.br (M.A.C.); rosemeire.pietro@unesp.br (R.C.L.R.P.); 3 Research Institute of Brewing and Malting, 614 00 Brno, Czech Republic

**Keywords:** *Achyrocline satureioides*, hydroalcoholic extract, natural bioactive compounds, flavonoids, antimicrobial, cosmetic emulsion, fragrance allergens, HaCaT, cytotoxicity

## Abstract

*Achyrocline satureioides* is a South American herb used in traditional medicine to treat a wide range of ailments. The healing and antimicrobial effects of this plant have already been covered by many studies, which have confirmed its beneficial effects on human health. In this study, the antimicrobial effect of *A. satureioides* hydroalcoholic extract against *Escherichia coli* ATCC10536, *Staphylococcus aureus* ATCC25923, *Staphylococcus epidermidis* ATCC12228 and *Lactobacillus acidophilus* INCQS00076 was determined. The cytotoxicity of the extract was tested on human HaCaT keratinocytes showing very favourable effects on the proliferation and renewal of keratinocytes. According to the results of the HPLC and GC-MS analyses, the lyophilized extract contained only a minimal amount of fragrance allergens. The extract was then used in two cosmetic formulations, and one of them showed a significant synergistic interaction with other cosmetic components. We suggest the use of *A.satureioides* hydroalcoholic extract as a suitable antimicrobial component of natural origin for cosmetic preparations as a substitute for commonly used preservatives that can cause skin irritation and as a material with its own biological activity.

## 1. Introduction

Natural extracts that exhibit antimicrobial activity are possible replacements for preservative substances added to cosmetic products. Some preservative substances are prohibited from being added to the cosmetic products due to their negative effects on human health or their potential to cause allergies. In the EU, the presence of such substances is regulated by the European Commission [1].

South America is home to a large variety of plant species that, traditionally, have many applications, e.g., in the production of dyes, drugs, antidotes, medicines, and foods [2]. *Achyrocline satureioides* is a native plant of South America, known by its common names “macela” or “marcela”, that grows in stony or sandy soils up to 3900 m above sea level [3,4]. Its golden yellow blossoms are the part of the plant most frequently used in native medicine [5].

Previously published studies have confirmed the antioxidant, antimicrobial, anti-inflammatory, and photoprotective effects of macela extracts [6,7,8]. Some other properties, such as sedative [9], hepatoprotective [10], antioxidant [11], immunomodulatory [12], antitumoural [13], and antiviral [14] properties, have also been attributed to this plant. Macela is traditionally used as a treatment for gastrointestinal disorders, digestive agent and antipyretic agent in infusions, macerate in cold water, decoction, syrup [9] and ingredient in the liquor industry [15]. Bidone et al. (2014) found that nano-emulsions prepared from both lyophilized and liquid extracts of *A. satureioides* via spontaneous emulsification provide a promising opportunity for the treatment of various skin disorders [16].

Phytochemical studies of *A. satureoides* have found that the antimicrobial and antioxidant activity of macela extract depends on the extract’s composition. The amount of compounds present depends on the extraction method due to the difference in dry extract yield, flavonoid profile, and the amount of free quercetin [2,17,18]. The majority of studies mostly present methods using ethanol extracts of the aerial parts of the plant [19,20,21].

**Figure 1:**
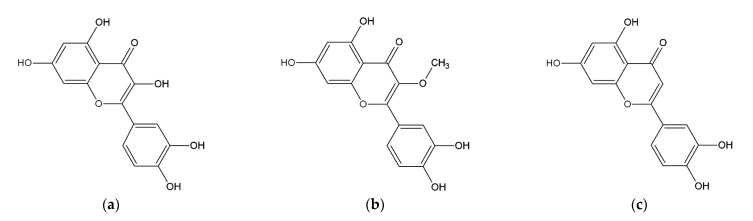
Main phenolic compounds present in *A. satureioides*: (**a**) quercetin, (**b**) 3-O-methylquercetin, (**c**) luteolin.

The main compounds responsible for the active principles were determined to be flavonoids. Flavonoids are antioxidants that are able to absorb free radicals of reactive oxygen species (ROS) and thereby prevent damage to the skin cells [22]. Antioxidants stimulate collagen synthesis and regulate gene expression, thereby slowing skin aging, improving skin hydration, helping with pigmentation disorders, and inhibiting lipid oxidation in sebum [23]. The results of the phytochemical analysis of extracts of *A. satureioides* identified quercetin, 3-O-methylquercetin, and luteolin as the main flavonoids present (Figure 1) [24,25]. Further investigations confirmed that these compounds are not toxic [26]. Additionally, the anticancer properties of quercetin and luteolin were evaluated in numerous studies focusing on many types of cancer: bladder [27,28], glioma [29], colon [30,31], liver [32,33], stomach [34,35], breast [36,37], skin [38,39] and lung cancer [40,41]. Quercetin is the most abundant flavonoid in the human diet. It has been described as inhibiting lipid peroxidation by scavenging reactive oxygen species. Quercetin has higher antioxidant activity compared to other flavonoids present in the *A. satureioides* extracts [36,38]. Several lyophilized ethanol extract fractions were shown to be more effective than the isolated components, suggesting that a possible synergy may exist, and thus their antioxidant properties are increased [7,42].

In this study, the antimicrobial effect of *A. satureioides* hydroalcoholic extract against particular bacterial strains was studied. As target strains were mainly selected bacteria causing human skin disorders. Furthermore, the cytotoxicity of the extract was tested on human HaCaT keratinocytes. The presence and composition of allergens were measured using chromatography. The application of the extract in two cosmetic products as a substitute for commonly used preservatives was also tested. To our best knowledge, this application of the plant extract has never been tested before.

## 2. Results and Discussion

### 2.1. Extract Characterization

The content of total phenols in the extract was 219.7 ± 5.0 mg GAE/g of the lyophilized extract, this value complies with the results reported by Guss et al. (2017), who determined the amount of phenolic substances as 231.8 ± 9.7 mg GAE/g extract in ethanolic macerate using the same method [43]. The flavonoid content in the extract was 122.51 ± 2.64 mg CE/g of lyophilized extract. The results were thus reported as catechin equivalents. Guss et al. (2017) reported a lower value of flavonoids present, specifically 43.56 ± 1.4 mg/g of extract, but in their study, a different assay was used to determine the flavonoid content [43]. Other authors using similar extraction conditions determined the content of flavonoids via ultra-fast liquid chromatography. The final content of total flavonoids was 1 mg/mL, which corresponds to 13 mg of phenolic substances per 1 g of dried plant material [44,45]. In our case, the flavonoid content in the extract was similar, specifically 8.13 ± 0.09 mg CE/g of dried plant material.

The antioxidant activity of the extract was determined using the Trolox equivalent antioxidant capacity (TEAC) assay. The antioxidant activity was 3.91 ± 0.07 mMol of Trolox equivalent per 100 g of dry weight of inflorescences. Similar results were presented by Goltz et al. (2018), where the antioxidant activity in the case of ethanolic extract of *A. satureioides* ranged between 6.62 and 15.15 mMol of Trolox equivalent per 100 g of dry weight of plant material depending on extraction time. The higher antioxidant activity in comparison with our results was probably caused by the use of ultrasound extraction [46].

### 2.2. Determination of Allergens Content by HPLC

HPLC analysis with a reversed phase was performed according to Villa et al. (2007) [47] to determine the allergen content of the extract because of its intended use in cosmetics emulsions. The amount of fragrance allergens in *A. satureioides* extract, required by EU legislation [1] to be declared on the label of cosmetic products, was determined. The amount of fragrant allergenic substances detected in the extract using this method was below the EU legislative limit.

### 2.3. Determination of Allergens Content by GC-MS

**Figure 2:**
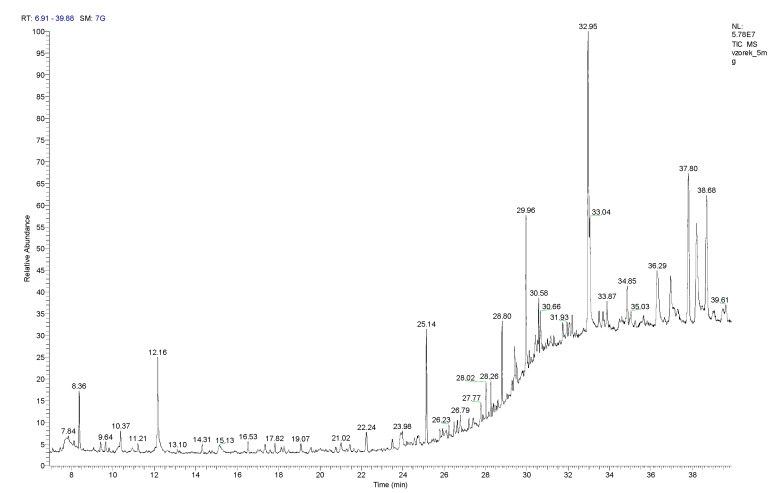
GC–MS chromatogram of *A. satureioides* extract (5 mg).

The chemical composition of the volatile compounds of 5 mg of the lyophilized hydroalcoholic extract was determined by GS/MS (Figure 2). The extract contained linalool (Rt = 8.36 min), which comprised 143.3 ± 17.6 µg per gram of the lyophilized extract, and eugenol (Rt = 23.49 min), which comprised 12.0 ± 2.0 µg per gram of extract. The amounts of these substances present in the lyophilized hydroalcoholic extract are significantly below the EU legislative limit for fragrance allergens present in leave-on cosmetic products, e.g., emulsions (Table 1).

The other fragrance substances found in the extract were caryophyllene (Rt = 9.40 min) 15.8 ± 0.9 µg; menthol (Rt = 10.24 min) 41.3 ± 0.8 µg; terpineol (Rt = 11.20 min) 10.0 ± 1.6 µg; carvone (Rt = 12.16 min) 263.8 ± 36.6 µg; anethole (Rt = 14.31 min) 22.9 ± 4.4 µg and vanillin (Rt = 29.43 min) 91.1 ± 3.2 µg per gram of lyophilized extract.

GC-MS chromatograms of Allergen Mix 1 and Allergen Mix 2 are shown in Figures S1 and S2, and their compositions are listed in Tables S1 and S2. The chemical compositions of the volatile compounds of 10 and 25 mg of the lyophilized hydroalcoholic extract are shown in Figures S3 and S4, and their compositions are listed in Tables S3 and S4.

### 2.4. MTT Cytotoxicity Assay

**Figure 3:**
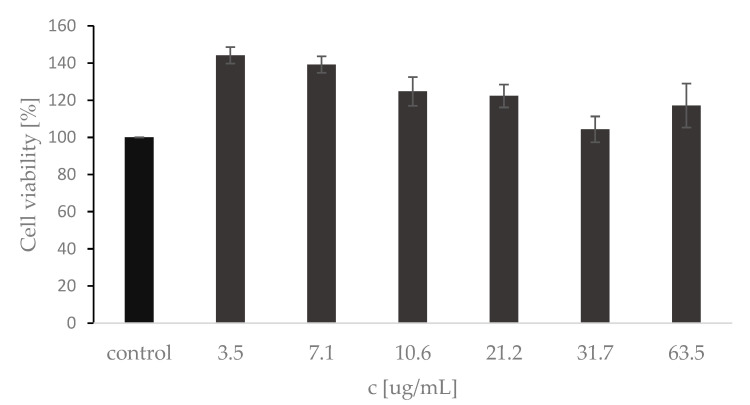
MTT assay of HaCaT cells: the control value represents mean ± SD for triplicate tests of the growth control with no extract. Results are expressed as the percentage of the control.

Cell viability assay using HaCaT cell line is considered to be a reliable model for assessing skin disorders and the biocompatibility and safety of various substances. HaCaT cells were exposed to the *A.satureioides* extract in the concentration range of 3.5–63.5 μg · mL^−1^ for 24 h. As shown in Figure 3, the extract did not induce cytotoxicity but increased the cell viability of keratinocytes when compared to the control in the tested concentration range of 3.5–63.5 μg·mL^−1^. An MTT cytotoxicity test with 24 h incubation in medium and extract mixture was used by other authors, who proposed a nanoemulsion of *A. satureioides* extract for wound healing [44]. Although cell viability dynamics were observed depending on the extract concentration, the incubation time was sufficient to assess whether the metabolic activity and proliferative capacity of HaCat cells is positively or negatively altered by the extract present. In our case, compared to the literature [44], the tested concentrations were even higher but still showed increased cell viability.

The viability of human cells was also determined using an enzymatic LDH test. The level of released LDH enzyme (units·mL^−1^) from damaged cells that catalyzes the interconversion of pyruvate and lactate was spectrophotometrically measured at 540 nm. The results were in accordance with the cell viability assay (MTT assay), and the extract did not induce cytotoxicity. LDH levels of extracts were compared with growth control (CC), where the LDH value of CC was determined at 461.30 ± 2.77 units·mL^−1^. Positive control (PC) with 10% ethanol reached LDH value at 836.67 ± 13.89 units·mL^−1^. All LDH values of tested samples at a concentration range of 3.5–63.5 μg·mL^−1^ did not achieve PC results. The highest LDH level was obtained at concentration 63,5 μg·mL^−1^ with an LDH value of 618.33 ± 3.33 units·mL^−1^.

### 2.5. Determination of MIC and MBC by Broth Microdilution Method

**Figure 4:**
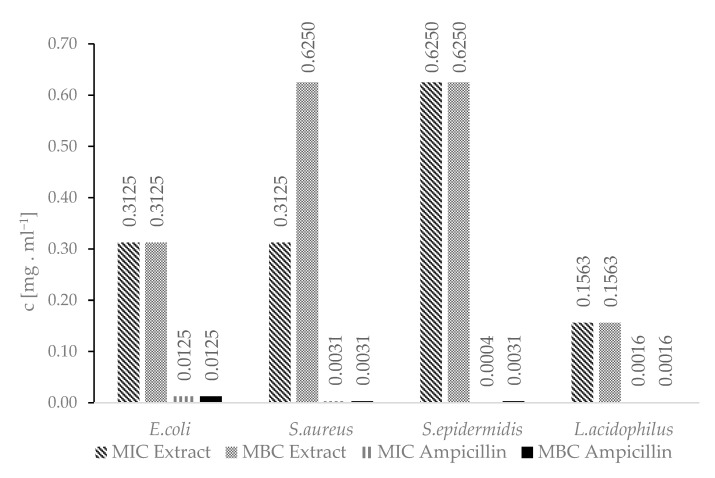
MIC and MBC concentrations of the hydroalcoholic extract.

The *A.satureioides* hydroalcoholic extract showed antimicrobial activity against all tested bacteria strains. The antimicrobial activity of the extract has already been covered by a large number of authors [48,49]. Lemos et al. (2000) investigated the antimicrobial activity of aqueous and other extracts against *E.coli* and *S.aureus* [49]. The antimicrobial activity of these extracts was confirmed at concentrations ranging from units to hundreds of micrograms per millilitre, which is consistent with our results (Figure 4). Ampicillin is used as a reference antibiotic in the methodology to determine the values of MIC and MBC established in accordance with the Guidelines of the Institute of CLSI-Clinical and Laboratory Standards M7-A11 for bacteria [50].

To our knowledge, no other studies have been designed to determine the antimicrobial activity of the hydroalcoholic extract of *A. satureioides* against lactobacilli. With an intention to add the extract to cosmetic products, we tested the antimicrobial activity of the extract against *Lactobacillus acidophilus* to determine if it is also suitable for cosmetic products for feminine hygiene. However, due to its strong antimicrobial activity against *L. acidophilus*, the hydroalcoholic extract of *A. satureioides* is not suitable for this purpose.

### 2.6. Determination of Inhibition Haloes by Agar Diffusion

**Figure 5:**
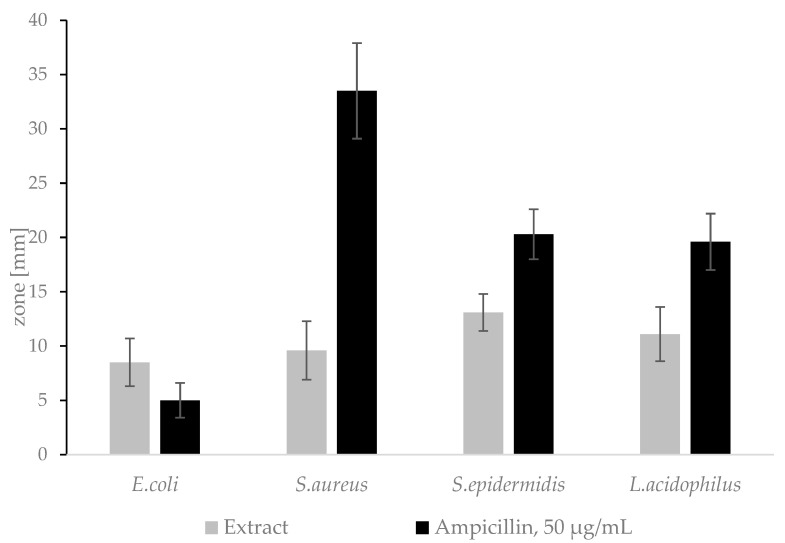
Inhibition zones of the extract on agar plates.

The *A.satureioides* extract showed antimicrobial activity against all tested bacteria strains (Figure 5). Ampicillin is used as a reference antibiotic in accordance with the Institute of CLSI-Clinical and Laboratory Standards M02-A13 Guideline [51]. The results comply with the previously published literature, where the authors proved the antimicrobial activity of freeze-dried hydroalcoholic extract against Gram-negative and Gram-positive bacteria, including *S.aureus* [52].

The hydroalcoholic extract of *A. satureioides* is not suitable to use in cosmetic products for feminine hygiene as confirmed by both the diffusion test and the broth dilution method.

### 2.7. Cosmetic Emulsions

**Figure 6:**
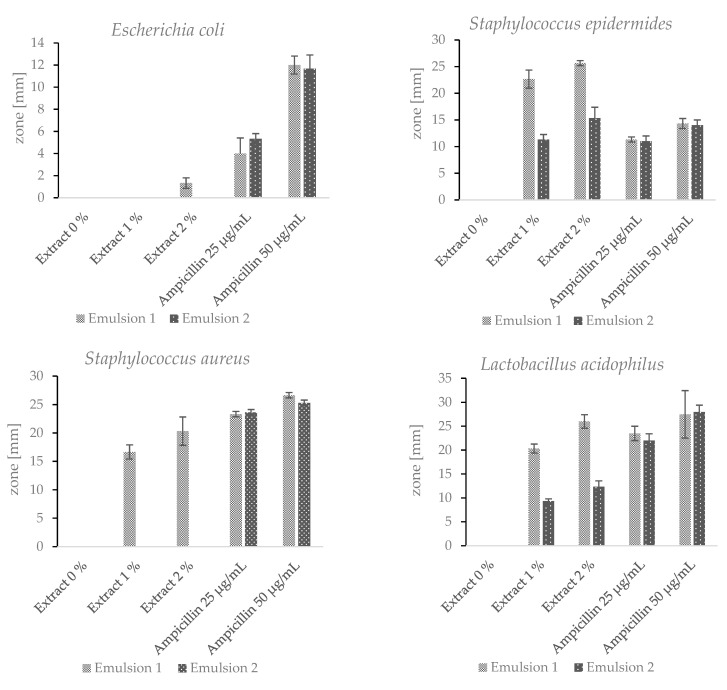
Inhibition zones of Emulsions 1 and 2 with 0%, 1% and 2% (*w*/*w*) of the extract.

Two emulsions were prepared with the addition of the lyophilised hydroacoholic extract (1% and 2% *w*/*w*) of *A.satureioides,* and these emulsions had different emulsifying components. Other components were identical for both emulsions. Despite this, the antimicrobial activity of the two emulsions was significantly different. Emulsion 1 showed antimicrobial activity against all tested bacteria (Figure 6). Emulsion 2 showed antimicrobial activity against *S. epidermidis* and *L.acidophilus,* but the inhibition zones were significantly smaller. Both of the emulsions without the added extract did not show any antimicrobial effect. As mentioned above, ampicillin is used as a reference antibiotic [51]. For a better comparison of the antimicrobial effects of the emulsions with the addition of the extract and ampicillin, the antibiotic was utilized in two different concentrations. The results presented in Figure 6 refer to the antimicrobial activity of the hydroalcoholic extract of the *Achyrocline satureioides* inflorescences from Brazil. The hydroalcoholic extract of the *Achyrocline satureioides* inflorescences originating in Paraguay displayed a comparable antimicrobial activity. The zone diameter varied by 5–10% in comparison with the Brazilian sample. Such variation does not even exceed the relative standard deviations for the majority of the tested bacterial strains.

Emulsion 1 contained the ingredient Emulfeel^®^ SGP, which consists of three components. Each Emulfeel^®^ SGP component shows antimicrobial or antiseptic activity. The antimicrobial properties of *Helianthus Annuus* (sunflower) seed oil were investigated by Aboki et al. (2012), who found that extracted oil showed antibacterial activity against *E.coli* and *S.aureus* [53]. Xylityl sesquicaprylate is used in the cosmetic products as a surfactant, emulsifier and antiseptic compound [54]. Polyacrylic acid is an emulsion stabilizer, and many authors investigated its antimicrobial activity in copolymers. The antimicrobial activity of the copolymers increased with increasing acrylic acid concentration [55]. Despite the antimicrobial properties of the individual components of Emulfeel^®^ SGP, no inhibition zone was observed in the case of the pure emulsion without the addition of the extract. It can therefore be concluded that the Emulfeel^®^ SGP components have a synergistic effect with the phenolic substances contained in the extract. Thus, the antimicrobial activity of the emulsion is significantly increased. The *A. satureioides* hydroalcoholic extract could be used as a safe hypoallergenic natural preservative, or as a substance to reduce the amount of other preservative ingredients present, while maintaining antimicrobial activity. However, more extensive tests need to be carried out, which goes beyond the scope of this study.

## 3. Materials and Methods

### 3.1. Plant Material

To determine the antimicrobial activity of the hydroalcoholic extract of *A. satureioides*, the used plant material was obtained via a scientific cooperation agreement signed between the School of Pharmaceutical Sciences of Araraquara (FCFAr-UNESP), Araraquara, São Paulo, Brazil, and the Pluridisciplinary Center for Chemical, Biological and Agronomic Research (CPQBA-UNICAMP), Campinas, São Paulo, Brazil. The obtained species *A. satureioides* was collected in the city of Campinas-SP at coordinates 22°48’ S; 47°07’ W. It was registered and authorized for use by SISGEN under the registration number AD73F75. The antimicrobial activity of the hydroalcoholic extract of *A. satureioides* was confirmed with plant material commercially purchased from Diochi spol. s r.o. (Prague, CZ) and obtained from Paraguay. The extract was subsequently characterized and tested for the presence of fragrance allergens and cell cytotoxic activity. Only plant material from Paraguay was available via the free market in CZ, likely due to strict rules about exporting plant material outside of Brazil [56].

### 3.2. Plant Extract Preparation

The preparation was carried out with 50 g of air-dried inflorescences of *A. satureioides,* which were mechanically ground and macerated in 1000 mL of hexane. In this step, the lipid fraction was removed to increase the water solubility of the final extract and efficiently extract active compounds. The mixture was tempered at 40 °C for 24 h, and the macerate was filtered. The plant material was used for the three-step maceration. In the first step, the plant material was tempered at 40 °C for 24 h in 500 mL of 70% EtOH; in the second and the third steps, the plant material was tempered at 40 °C. In each step, the macerate was filtered, and the ethanol was completely removed via rotary evaporation under reduced pressure.

The freeze-dried extract (FDE) was prepared using Edwards Modulyo (BOC Edwards, Tonawanda, NY, USA) freeze-drying equipment. The extract was freeze-dried under a controlled temperature (−60 °C) and pressure (−10^−2^ bar). The final dry extract powder consisted of all three ethanolic extracts and was stored in a freezer (−20 °C) until further use.

The lyophilized extract from commercially purchased plant material was prepared in the same way using FreeZone Triad (Labconco, Kansas City, MI, USA) freeze-drying equipment.

### 3.3. Antioxidant Activity

The antioxidant activity of the hydroalcoholic extract was measured using the Trolox equivalent antioxidant capacity (TEAC) assay [57]. The ABTS·+ solution was prepared by diluting 7 mM ABTS (2,2′–azino–bis(3–ethylbenzothiazoline–6–sulfuric acid) diammonium salt) (Sigma–Aldrich, Burlington, MA, USA) and 2.45 mM potassium persulphate (Sigma–Aldrich, USA) in deionized water. The solution was stored in a dark place at room temperature for 12–16 h before use. The blank solution was prepared by diluting the ABTS·+ solution with UV–VIS ethanol to a final absorption value: A_734 nm_ = 0.70 ± 0.02. The antioxidant activity of the hydroalcoholic extract was measured by adding 10 μL to 1 mL of diluted ABTS·+, and the decrease in absorption was measured. The Trolox calibration standards were prepared in the range of concentration 0.2–1.6 μmol·mL^−1^. The absorbance of the samples was measured in triplicate.

### 3.4. Analysis of Total Phenols

The amount of total phenols present in the hydroalcoholic extract was measured by the Folin–Ciocalteu assay [58]. The solution of 1 mL Folin–Ciocalteu reagent (Penta Chemicals, CZ, 10 times diluted), 1 mL of deionized water and 50 μL of the hydroalcoholic extract was prepared, mixed and incubated for 5 min at room temperature. The blank solution was prepared with 50 μL of deionized water. Saturated Na_2_CO_3_ solution (1 mL) was added to the measured solutions, which were incubated for 15 min at room temperature. The absorbance of solutions was measured at a wavelength of 750 nm. The calibration standards of gallic acid (Sigma–Aldrich, USA) were prepared in the range of concentrations: 0.1–0.7 mg · mL^−1^. The absorbance of the samples was measured in triplicate, and the results are expressed as mg of gallic acid equivalent (GAE)/g of each sample.

### 3.5. Analysis of Total Flavonoids

The amount of total flavonoids present in the extract was measured using a flavonoid colorimetric assay [59]. A solution of 0.5 mL hydroalcoholic extract, 1.5 mL deionized water, and 0.2 mL 5% NaNO_2_ (Lach-Ner, CZ) was prepared, mixed and incubated for 5 min at room temperature. In the case of a blank solution, the 0.5 mL of extract was replaced with the same amount of deionized water. Then, 0.2 mL of 10% AlCl_3_ (Lach-Ner, CZ) solution was added, mixed and incubated at room temperature for 5 min. Then, 1.5 mL of 5% NaOH (Lach-Ner, CZ) solution and 1.0 mL of deionized water were added, mixed and incubated for 15 min at room temperature. The absorbance was measured at a wavelength of 510 nm. The calibration standards of catechin (Sigma–Aldrich, USA) were prepared in a range of concentrations: 0.05–0.3 mg · mL^−1^. The absorbance of the samples was measured in triplicate, and the results were expressed as milligram of catechin equivalent (CE) per gram of dry weight (mg CE/g dw) of sample.

### 3.6. Determination of Allergen Content by HPLC-DAD

Reversed-phase HPLC analysis was performed according to Villa et al. (2007) [47] to determine the fragrance allergen content of the extract. The analysis was performed in triplicate using HPLC/PDA Dionex UltiMate 3000 (Thermo Fischer, Waltham, MA, USA), DAD detector Vanquish series (Thermo Fischer, USA), column YMC-Triart C18 ExRS S-3µm, 8nm Analytical HPLC Column, 150 × 4.6 mm. Allergen standards were obtained from Accustandard Inc. (New Haven, CT, USA).

### 3.7. Determination of Allergen Content by GC-MS

The major volatile compounds of the lyophilized hydroalcoholic extract of *Achyrocline satureioides* were analysed using a Trace™ 1310 gas chromatograph with the TSQ 9000 mass detector (Thermo Fisher Scientific Inc., Waltham, MA, USA) and the Supelcowax capillary column (30 m × 0.25 mm; I.D. 0.25 μm, Supelco, Bellefonte, PA, USA). Helium was used as carrying gas at a flow rate of 1 mL·min^−1^.

The lyophilized extract material was extracted in amounts of 5, 10 and 25 mg using ethyl acetate for 10 min, and then filtered through a nylon filter (0.22 μm). One microliter of the sample was injected onto the column (splitless mode, 1 min). The temperature of the injector was 250 °C, and the temperature of the transfer line was 220 °C. The conditions of the analysis were as follows: incubation at 70 °C for 1 min, followed by temperature increase up to 135 °C (10 °C/min), and the temperature was maintained at 135 °C for 2 min. The temperature was increased up to 170 °C (3 °C/min) and maintained for 1 min. This was followed by an increase in temperature to 250 °C (10 °C/min.), where the temperature was maintained for 20 min. The TSQ 9000 MS detector was used in electron ionization mode and set at 70 eV, ion source temperature: 250 °C, scanning range: 35–350 *m*/*z* and scanning speed: 0.2 s.

### 3.8. Cell Culture and Treatment

The assay was performed with human epidermal keratinocytes (HaCaT), and the cells were obtained from CLS Cell Lines Service GmbH (Eppelheim, Germany). The cells were cultured in Dulbecco’s Modified Eagle Medium (DMEM) (Lonza Biotec, CZ, USA) without sodium pyruvate, supplemented with high glucose, 0.4 mM L-glutamine, 1% penicillin/streptomycin/amphotericin B solution (Antimycotin-Antibiotic 100×, Sigma-Aldrich, Burlington, MA, USA), and 10% of heat-inactivated fetal bovine serum (FBS). HaCaT cells were incubated in a humidified 5% (v/v) CO_2_ atmosphere at 37 °C for 24 h. The lyophilized extract was dissolved in 70% EtOH. Samples were diluted with DMEM up to a concentration range of 3.5–63.5 μg·mL^−1^. The control was DMEM medium, and 10% ethanol was used as a negative control. After 24 h of incubation in a humidified 5% (*v*/*v*) CO_2_ atmosphere at 37 °C, cell viability was determined [60].

### 3.9. MTT Assay

Cell viability was assessed using a colorimetric MTT reduction assay to determine cell metabolic activity. Following the treatment, 20 μL of MTT dissolved in PBS (2.5 mg·mL^−1^) was added to each sample and incubated for 3 h in humidified 5% (*v*/*v*) CO2 atmosphere at 37 °C, and then 100 μL of 10% SDS in PBS was added to each well. Plates were stored in the dark and evaluated the next day using ELISA Reader at 562 nm [61].

### 3.10. Microorganisms

The following reference strains were used: *Escherichia coli* ATCC10536, *Staphylococcus aureus* ATCC25923, *Staphylococcus epidermidis* ATCC12228, and *Lactobacillus acidophilus* INCQS00076. The bacteria were transferred to a fresh brain infusion medium (BHI) and allowed to grow aerobically for 24 h at 37 °C. The inoculums for the assays were made by diluting bacterial suspensions up to McFarland standard turbidity of 0.5 (10^8^ CFU·mL^−1^) [50], except for *L. acidophilus*, which was diluted to 5.0 McFarland standard (10^9^ CFU·mL^−1^) [62].

### 3.11. Broth Microdilution Method

Minimal inhibitory concentrations (MIC) were established in accordance with the Guidelines of the Institute of Clinical and Laboratory Standards M7-A11 for bacteria [50]. The test was performed on 96-well sterile microplates. The extract in DMSO solution (DMSO:medium, 1:10 *v*/*v*) was transferred to wells of microplates in order to obtain double serial dilutions in a concentration range of 2.5 to 0.0012 mg·mL^−1^. Each microplate contained an antibiotic control, ampicillin (Sigma Chemical Co., Burlington, MA, USA), with concentrations ranging from 12.5 to 0.006 μg·mL^−1^. An inoculum containing 5 × 10^6^ CFU·mL^−1^ of bacteria in BHI broth (the final concentration in the well was 2.5 × 10^6^ CFU·mL^−1^) was added to each well. A number of wells on each plate belonged to control of sterility of media, the viability of inoculum, the sterility of the extract solution, and controls to exclude the inhibitory effect of DMSO. The plates were prepared in triplicates and aerobically incubated for 24 h at 37 °C. After incubation, the 0.5% aqueous resazurin solution was added to each well in order to observe the viability of bacteria.

The lowest extract concentration that prevented growth was determined to be MIC. By sub-cultivating microplate wells on Müller Hinton agar plates (MHA), which were aerobically incubated for 24 h at 37 °C, the minimal bactericidal concentration (MBC) was determined.

### 3.12. Method of Agar Diffusion

According to the Institute of Clinical and Laboratory Standards’ M02-A13 Guideline, the test for bacteria was performed on Müller Hinton agar (MHA) [51]. Each plate was seeded separately with 1% (*v*/*v*) bacterial cell suspension (10^6^ CFU·mL^−1^). A 1% (*v*/*v*) *L. acidophilus* bacterial cell culture containing 10^7^ CFU · mL^−1^ was inoculated on MRS agar [51]. Next, 100 μL of the extract at a concentration of 5.0 mg·mL^−1^ in dimethyl sulfoxide in saline solution (saline:DMSO, 5:1 *v*/*v*) was added to the agar plate. Each plate contained ampicillin (50 μg·mL^−1^) and DMSO solvent control.

### 3.13. Preparation of Cosmetic Emulsions

Two emulsions with different ingredients and two temperatures were prepared, and their compositions are shown in Table 2 and Table 3. The lyophilized hydroalcoholic extract of *Achyrocline satureioides* was added to prepared emulsions at room temperature at two concentrations: 1% *w*/*w* and 2% *w*/*w*.

### 3.14. Antimicrobial Activity of Emulsions

According to the Institute of Clinical and Laboratory Standards’ M02-A13 Guideline, the test for bacteria was performed on Müller Hinton agar (MHA) [51]. Plates were seeded separately with each 1% (*v*/*v*) bacterial cell suspension (10^6^ CFU·mL^−1^). A 1% (*v*/*v*) *L. acidophilus* bacterial cell culture containing 10^7^ CFU·mL^−1^ was inoculated on MRS agar [51]. Each agar plate contained 100 µL of an emulsion with 0%, 1% and 2% additions of the extract, ampicillin (25 and 50 μg·mL^−1^). Both emulsions were tested in triplicate for all bacteria.

### 3.15. Statistical Analysis

All experiments were performed in triplicates. Results are presented as mean ± standard deviation (SD). For hypothesis testing, one-way ANOVA was used and set at a significance level of 0.05. Data were evaluated using Tukey’s HSD test.

## 4. Conclusions

The antimicrobial and antioxidant activity of the hydroalcoholic extract confirmed the results of the previously published studies. Antimicrobial activity against *L. acidophilus* was determined. Unfortunately, due to the strong antimicrobial activity of the extract against *L. acidophilus*, the use of this extract in feminine hygiene products is not recommended. It was found that, in a suitable combination with other components of the emulsion formula, the antimicrobial activity against the tested bacteria increased. Thus, it could be possible to limit the amount of preservatives necessary to maintain the microbiological stability of the cosmetic product. Moreover, the high antimicrobial activity of *A. satureioides* extract can be considered an additional biological activity. In addition, the hydroalcoholic extract of *A. satureioides* did not show a toxic effect on keratinocytes; on the contrary, very favourable effects on cell proliferation and renewal were observed. HPLC analysis also confirmed that the extract does not contain any fragrance allergens, which significantly reduces the likelihood of a possible allergic reaction to this component of the cosmetic product. A very low amount of fragrance allergens was detected via GC-MS analysis, but these concentrations are insignificant from a legislative point of view. In conclusion, the hydroalcoholic extract of *A. satureioides* could serve as a safe hypoallergenic natural preservative for use in cosmetic products. This application of the plant extract has never been tested before.

## Figures and Tables

**Table 1:** The amount of fragrance substances present in lyophilized hydroethanolic extract of *A. satureioides* and in cosmetic emulsions.

Fragrance Substance	Rt (Min)	Amount of Substance (µg Per Gram of Lyophilized Extract)	Amount of Substance (µg Per Gram of Emulsion with 2% of Lyophilised Extract)	EU Fragrance Allergen Limit (µg Per Gram of Emulsion) *
Linalool	8.36	143.3 ± 17.6	2.9 ± 0.4	10.0
Caryophyllene	9.40	15.8 ± 0.9	0.3 ± 0.0	n.r.
Menthol	10.24	41.3 ± 0.8	0.8 ± 0.0	n.r.
Terpineol	11.20	10.0 ± 1.6	0.2 ± 0.0	n.r.
Carvone	12.16	263.8 ± 36.6	5.3 ± 0.7	n.r.
Anethole	14.31	22.9 ± 4.4	0.5 ± 0.1	n.r.
Eugenol	23.49	12.0 ± 2.0	0.2 ± 0.0	10.0
Vanillin	29.43	91.1 ± 3.2	1.8 ± 0.1	n.r.

n.r.—EU legislation does not require the amount of fragrances present to be listed on the packaging. * When the amount of substance present is higher than the limit, its listing on the packaging is required by EU legislation.

**Table 2:** Composition of Emulsion 1.

Name of Ingredient	INCI Name of Ingredient	*w*/*w* (%)
Emulfeel^®^ SGP	*Helianthus Annuus* (sunflower) seed oil (and) polyacrylic acid (and) xylityl sesquicaprylate	5.00
Octyl stearate	Ethylhexyl stearate	1.00
Grape seed oil	*Vitis Vinifera* seed oil	1.00
Butylhydroxytoluene	BHT	0.02
Glicerine	Glycerin	3.00
Purified water	Water	Up to 100%

The emulsion was prepared via mixing all components at 22 °C.

**Table 3:** Composition of Emulsion 2.

Name of Ingredient	INCI Name of Ingredient	*w*/*w* (%)
Polawax™ NF	Cetylstearyl alcohol (and) polysorbate 60.	10.00
Octyl stearate	Ethylhexyl stearate	1.00
Grape seed oil	*Vitis Vinifera* seed oil	1.00
Butylhydroxytoluene	BHT	0.02
Glicerine	Glycerin	3.00
Purified water	Water	Up to 100%

The emulsion was prepared by mixing all components at 80 °C.

## Data Availability

Not applicable.

## Supplementary Materials

The following supporting information can be downloaded at: https://www.mdpi.com/article/10.3390/plants12102027/s1, Figure S1: GC-MS chromatogram Allergen Mix 1; Figure S2: GC-MS chromatogram Allergen Mix 2; Figure S3: GC-MS chromatogram of *A.satureioides* lyophilized extract (10 mg); Figure S4. GC-MS chromatogram of *A.satureioides* lyophilized extract (25 mg); Table S1: GC-MS Allergen Mix 1 composition; Table S2: GC-MS Allergen Mix 2 composition; Table S3: Fragrance allergen composition of *A.satureioides* lyophilized extract (10 mg); Table S4: Fragrance allergen composition of *A.satureioides* lyophilized extract (25 mg).

## References

[1] Publications Office of the European Union (2009). Regulation (EC) No 1223/2009 of the European Parliament and of the Council of 30 November 2009 on Cosmetic Products.

[2] Retta D., Dellacassa E., Villamil J., Suárez S.A., Bandoni A.L. (2012). Marcela, a promising medicinal and aromatic plant from Latin America: A review. Ind. Crop. Prod..

[3] Lorenzo D., Atti-Serafini L., Santos A.C., Frizzo C.D., Paroul N., Paz D., Dellacassa E., Moyna P. (2000). *Achyrocline satureioides* Essential Oils from Southern Brazil and Uruguay. Planta Med..

[4] De Souza K., Bassani V., Schapoval E. (2007). Influence of excipients and technological process on anti-inflammatory activity of quercetin and *Achyrocline satureioides* (Lam.) D.C. extracts by oral route. Phytomedicine.

[5] Davies P., Villamil J. (2004). Estudios en Domestication y Cultivo de Especies Medicinales y Aromáticas Nativas.

[6] Morquio A., Rivera-Megret F., Dajas F. (2005). Photoprotection by topical application of *Achyrocline satureioides* (‘Marcela’). Phytother. Res..

[7] Polydoro M., de Souza K., Andrades M., Da Silva E., Bonatto F., Heydrich J., Pizzol F.D., Schapoval E., Bassani V., Moreira J. (2004). Antioxidant, a pro-oxidant and cytotoxic effects of *Achyrocline satureioides* extracts. Life Sci..

[8] Arredondo M., Blasina F., Echeverry C., Morquio A., Ferreira M., Abin-Carriquiry J., Lafon L., Dajas F. (2004). Cytoprotection by *Achyrocline satureioides* (Lam) D.C. and some of its main flavonoids against oxidative stress. J. Ethnopharmacol..

[9] Simões C.M.O., Schenkel E.P., Bauer L., Langeloh A. (1988). Pharmacological investigations on *Achyrocline satureioides* (Lam.) DC., compositae. J. Ethnopharmacol..

[10] Kadarian C., Broussalis A., Miño J., Lopez P., Gorzalczany S., Ferraro G., Acevedo C. (2002). Hepatoprotective activity of *Achyrocline satureioides* (Lam) D. C. Pharmacol. Res..

[11] Desmarchelier C., Coussio J., Ciccia G. (1998). Antioxidant and free radical scavenging effects in extracts of the medicinal herb *Achyrocline satureioides* (Lam.) DC. (“marcela”). Braz. J. Med. Biol. Res..

[12] Cosentino M., Bombelli R., Carcano E., Luini A., Marino F., Crema F., Dajas F., Lecchini S. (2008). Immunomodulatory properties of *Achyrocline satureioides* (Lam.) D.C. infusion: A study on human leukocytes. J. Ethnopharmacol..

[13] Souza P., Bianchi S., Figueiró F., Heimfarth L., Moresco K., Gonçalves R., Hoppe J., Klein C., Salbego C., Gelain D. (2018). Anticancer activity of flavonoids isolated from *Achyrocline satureioides* in gliomas cell lines. Toxicol. Vitr..

[14] Bettega J.M.R., Teixeira H., Bassani V.L., Barardi C.R.M., Simões C.M.O. (2004). Evaluation of the antiherpetic activity of standardized extracts of *Achyrocline satureioides*. Phytother. Res..

[15] Guariniello J., Iannicelli J., Peralta P.A., Escandón A.S. (2018). In vivo and in vitro propagation of “macela”: A medicinal-aromatic native plant with ornamental potential. Ornam. Hortic..

[16] Bidone J., Zorzi G.K., Carvalho E.L., Simões C.M., Koester L.S., Bassani V.L., Teixeira H.F. (2014). Incorporation of *Achyrocline satureioides* (Lam.) DC extracts into topical nanoemulsions obtained by means of spontaneous emulsification procedure. Ind. Crop. Prod..

[17] Diaz C., Heinzen H. (2006). Variations in the flavonoid profile and free quercetin content in different extracts of *Achyrocline satureoides*. Acta Farm. Bonaer..

[18] Takeuchi T.M., Rubano M.L., Meireles M.A.A. (2010). Characterization and Functional Properties of Macela (*Achyrocline Satureioides*) Extracts Obtained by Supercritical Fluid Extraction Using Mixtures of CO_2_ Plus Ethanol. Food Bioprocess Technol..

[19] Casero C., Machín F., Méndez-Álvarez S., Demo M., Ravelo G., Pérez-Hernández N., Joseph-Nathan P., Estévez-Braun A. (2014). Structure and Antimicrobial Activity of Phloroglucinol Derivatives from *Achyrocline satureioides*. J. Nat. Prod..

[20] Kaloga M., Hänsel R., Cybulski E.-M. (1983). Isolierung eines Kawapyrons aus *Achyrocline satureioides*. Planta Med..

[21] Ferraro G.E., Norbedo C., Coussio J.D. (1981). Polyphenols from *Achyrocline satureioides*. Phytochemistry.

[22] Nimse S., Pal D. (2015). Free radicals, natural antioxidants, and their reaction mechanisms. RSC Adv..

[23] Baumann L. (2018). How to Use Oral and Topical Cosmeceuticals to Prevent and Treat Skin Aging. Facial Plast. Surg. Clin. N. Am..

[24] Salgueiro A.C., Folmer V., da Rosa H.S., Costa M.T., Boligon A.A., Paula F.R., Roos D.H., Puntel G.O. (2016). In vitro and in silico antioxidant and toxicological activities of *Achyrocline satureioides*. J. Ethnopharmacol..

[25] Ferraro G., Anesini C., Ouvina A., Retta D., Filip R., Gattuso M., Gattuso S., Hnatyszyn O., Bandoni A. (2008). Total phenolic content and antioxidant activity of extracts of *Achyrocline satureioides* flowers from different zones in Argentina. Lat. Am. J. Pharm..

[26] Duke J. (2002). Handbook of Medicinal Herbs.

[27] Kim Y., Kim W.-J., Cha E.-J. (2011). Quercetin-induced Growth Inhibition in Human Bladder Cancer Cells Is Associated with an Increase in Ca^2+^-activated K^+^ Channels. Korean J. Physiol. Pharmacol..

[28] Kilani-Jaziri S., Frachet V., Bhouri W., Ghedira K., Chekir-Ghedira L., Ronot X. (2011). Flavones inhibit the proliferation of human tumor cancer cell lines by inducing apoptosis. Drug Chem. Toxicol..

[29] Michaud-Levesque J., Bousquet-Gagnon N., Béliveau R. (2012). Quercetin abrogates IL-6/STAT3 signaling and inhibits glioblastoma cell line growth and migration. Exp. Cell Res..

[30] Kim H.-J., Kim S.-K., Kim B.-S., Lee S.-H., Park Y.-S., Park B.-K., Kim S.-J., Kim J., Choi C., Kim J.-S. (2010). Apoptotic Effect of Quercetin on HT-29 Colon Cancer Cells via the AMPK Signaling Pathway. J. Agric. Food Chem..

[31] Lim D.Y., Jeong Y., Tyner A.L., Park J.H.Y. (2007). Induction of cell cycle arrest and apoptosis in HT-29 human colon cancer cells by the dietary compound luteolin. Am. J. Physiol. Liver Physiol..

[32] Tan J., Wang B., Zhu L. (2009). Regulation of Survivin and Bcl-2 in HepG2 Cell Apoptosis Induced by Quercetin. Chem. Biodivers..

[33] Selvendiran K., Koga H., Ueno T., Yoshida T., Maeyama M., Torimura T., Yano H., Kojiro M., Sata M. (2006). Luteolin Promotes Degradation in Signal Transducer and Activator of Transcription 3 in Human Hepatoma Cells: An Implication for the Antitumor Potential of Flavonoids. Cancer Res..

[34] Wang P., Zhang K., Zhang Q., Mei J., Chen C.-J., Feng Z.-Z., Yu D.-H. (2012). Effects of quercetin on the apoptosis of the human gastric carcinoma cells. Toxicol. Vitr..

[35] Wu B., Zhang Q., Shen W., Zhu J. (2008). Anti-proliferative and chemosensitizing effects of luteolin on human gastric cancer AGS cell line. Mol. Cell. Biochem..

[36] Chou C.-C., Yang J.-S., Lu H.-F., Ip S.-W., Lo C., Wu C.-C., Lin J.-P., Tang N.-Y., Chung J.-G., Chou M.-J. (2010). Quercetin-mediated cell cycle arrest and apoptosis involving activation of a caspase cascade through the mitochondrial pathway in human breast cancer MCF-7 cells. Arch. Pharmacal Res..

[37] Kim M.J., Woo J.S., Kwon C.H., Kim J.H., Kim Y.K., Kim K.H. (2012). Luteolin induces apoptotic cell death through AIF nuclear translocation mediated by activation of ERK and p38 in human breast cancer cell lines. Cell Biol. Int..

[38] Kuo P.-C., Liu H.-F., Chao J.-I. (2004). Survivin and p53 Modulate Quercetin-induced Cell Growth Inhibition and Apoptosis in Human Lung Carcinoma Cells. J. Biol. Chem..

[39] Ruan J.-S., Liu Y.-P., Zhang L., Yan L.-G., Fan F.-T., Shen C.-S., Wang A.-Y., Zheng S.-Z., Wang S.-M., Lu Y. (2012). Luteolin reduces the invasive potential of malignant melanoma cells by targeting β3 integrin and the epithelial-mesenchymal transition. Acta Pharmacol. Sin..

[40] Zhang X., Huang S., Xu Q. (2004). Quercetin inhibits the invasion of murine melanoma B16-BL6 cells by decreasing pro-MMP-9 via the PKC pathway. Cancer Chemother. Pharmacol..

[41] Zhao Y., Yang G., Ren N., Zhang X., Yin Q., Sun X. (2011). Luteolin suppresses growth and migration of human lung cancer cells. Mol. Biol. Rep..

[42] De Souza K., Schapoval E., Bassani V. (2002). LC determination of flavonoids: Separation of quercetin, luteolin and 3-O-methylquercetin in *Achyrocline satureioides* preparations. J. Pharm. Biomed. Anal..

[43] Guss K.L., Pavanni S., Prati B., Dazzi L., de Oliveira J.P., Nogueira B.V., Pereira T.M., Fronza M., Endringer D.C., Scherer R. (2017). Ultrasound-assisted extraction of *Achyrocline satureioides* prevents contrast-induced nephropathy in mice. Ultrason. Sonochem..

[44] Balestrin L.A., Kreutz T., Fachel F.N.S., Bidone J., Gelsleichter N.E., Koester L.S., Bassani V.L., Braganhol E., Dora C.L., Teixeira H.F. (2021). *Achyrocline satureioides* (Lam.) DC (Asteraceae) Extract-Loaded Nanoemulsions as a Promising Topical Wound Healing Delivery System: In Vitro Assessments in Human Keratinocytes (HaCaT) and HET-CAM Irritant Potential. Pharmaceutics.

[45] Balestrin L.A., Back P.I., Marques M.D.S., Araújo G.D.M.S., Carrasco M.C.F., Batista M.M., Silveira T., Rodrigues J.L., Fachel F.N.S., Koester L.S. (2022). Effect of Hydrogel Containing *Achyrocline satureioides* (Asteraceae) Extract–Loaded Nanoemulsions on Wound Healing Activity. Pharmaceutics.

[46] Goltz C., Ávila S., Barbieri J.B., Igarashi-Mafra L., Mafra M.R. (2018). Ultrasound-assisted extraction of phenolic compounds from Macela (*Achyrolcine satureioides*) extracts. Ind. Crop. Prod..

[47] Villa C., Gambaro R., Mariani E., Dorato S. (2007). High-performance liquid chromatographic method for the simultaneous determination of 24 fragrance allergens to study scented products. J. Pharm. Biomed. Anal..

[48] Calvo D., Cariddi L.N., Grosso M., Demo M.S., Maldonado A.M. (2006). *Achyrocline satureioides* (LAM.) DC (Marcela): Antimicrobial activity on *Staphylococcus* spp. and immunomodulating effects on human lymphocytes. Rev. Latinoam. Microbiol..

[49] Lemos G., Oliviera L., Eberli B., Motta O., Folly M. (2000). Bactericidal activity of macela (*Achyrocline satureioides* (Lam.) DC.) and jaborandi-falso (*Piper aduncum* L.) against strains of *Staphylococcus aureus* isolated from subclinical bovine mastitis. Rev. Bras. Plantas Med..

[50] Clinical and Laboratory Standards Institute (2018). Methods for Dilution Antimicrobial Susceptibility Test for Bacteria That Grow Aerobically: Approved Standard M7-A11.

[51] Clinical and Laboratory Standards Institute (CLSI) (2018). Performance Standards for Antimicrobial Disk Susceptibility Tests.

[52] Moresco K.S., Silveira A.K., Zeidán-Chuliá F., Correa A.P.F., Oliveria R.R., Borges A.G., Grun L., Barbé-Tuana F., Zmozinski A., Brandelli A. (2017). Effects of *Achyrocline satureioides* Inflorescence Extracts against Pathogenic Intestinal Bacteria: Chemical Characterization, In Vitro Tests, and In Vivo Evaluation. Evid.-Based Complement. Altern. Med..

[53] Aboki M., Mohammed M., Musa S., Zuru B. (2012). Physicochemical and anti-microbial properties of sunflower (*Helianthus annuus* L.) seed oil. Int. J. Sci. Technol..

[54] Nogueira C., Mussi L., Baby A.R., Zupeli R., Magalhães W.V. (2023). Xylityl Sesquicaprylate Efficacy as an Antiseptic Ingredient for Oral Care Products (Mouthwash): An In Vitro Screening Investigation against Eight Microorganisms. Molecules.

[55] Gratzl G., Paulik C., Hild S., Guggenbichler J.P., Lackner M. (2014). Antimicrobial activity of poly(acrylic acid) block copolymers. Mater. Sci. Eng. C.

[56] (2015). Law, No. 13.123 of 20 May 2015: Access and Benefits Sharing of Genetic Resources and Associated Traditional Knowledge.

[57] Re R., Pellegrini N., Proteggente A., Pannala A., Yang M., Rice-Evans C. (1999). Antioxidant activity applying an improved ABTS radical cation decolorization assay. Free Radic. Biol. Med..

[58] Singleton V.L., Orthofer R., Lamuela-Raventós R.M. (1999). Analysis of total phenols and other oxidation substrates and antioxidants by means of folinciocalteu reagent. Oxid. Antioxid. Part A.

[59] Chang C.-C., Yang M.-H., Wen H.-M., Chern J.-C. (2002). Estimation of total flavonoid content in propolis by two complementary colometric methods. J. Food Drug Anal..

[60] Bokrova J., Marova I., Matouskova P., Pavelkova R. (2019). Fabrication of novel PHB-liposome nanoparticles and study of their toxicity in vitro. J. Nanopart. Res..

[61] Li X., Turánek J., Knötigová P., Kudláčková H., Masek J., Parkin S., Rankin S., Knutson B.L., Lehmler H.-J. (2009). Hydrophobic tail length, degree of fluorination and headgroup stereochemistry are determinants of the biocompatibility of (fluorinated) carbohydrate surfactants. Colloids Surf. B Biointerfaces.

[62] Ocaña V., Silva C., Nader-Macías M.E. (2006). Antibiotic Susceptibility of Potentially Probiotic Vaginal Lactobacilli. Infect. Dis. Obstet. Gynecol..

