# Supplementary material for: Achyrocline satureioides Hydroalcoholic Extract as a Hypoallergenic Antimicrobial Substitute of Natural Origin for Commonly Used Preservatives in Cosmetic Emulsions
Source: Plants (Basel). 2023 May 18;12(10):2027. doi: 10.3390/plants12102027 (PMC10222649; doi:10.3390/plants12102027)
Supplement: Supplementary file 1 [file plants-12-02027-s001.zip › plants-2375611-supplementary.pdf]

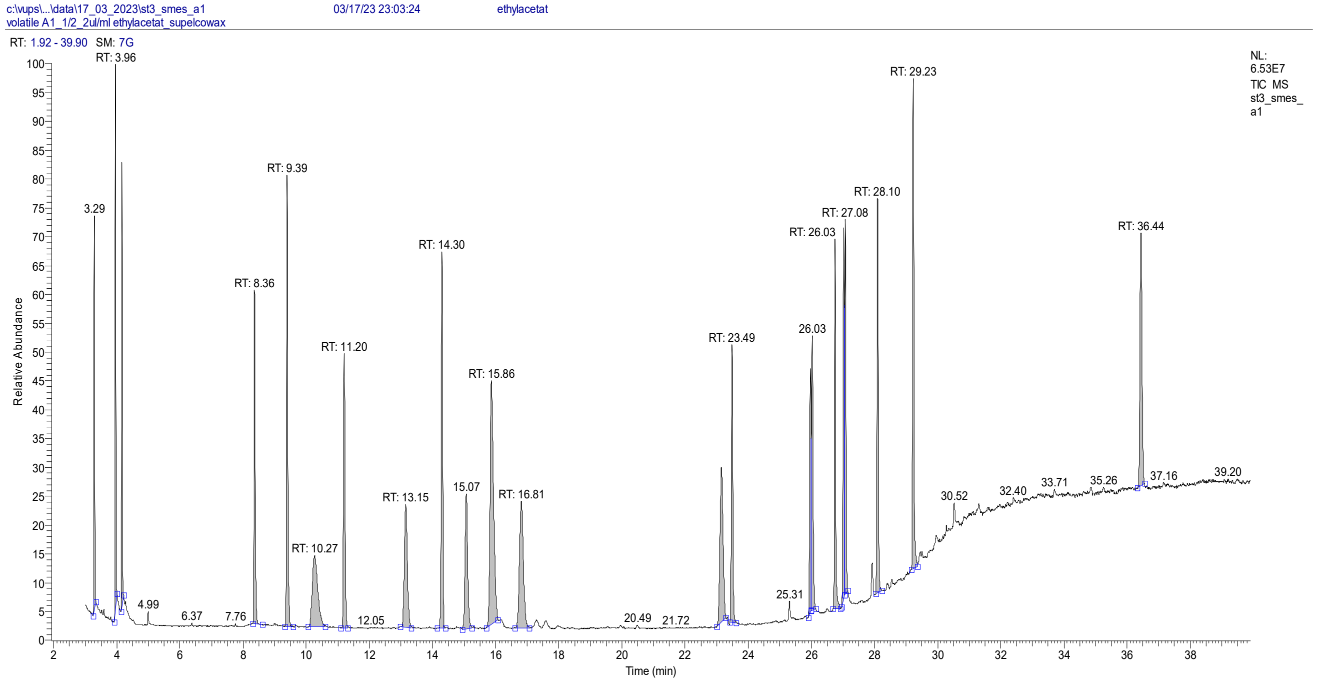

Figure S1. GC-MS chromatogram Allergen Mix 1.

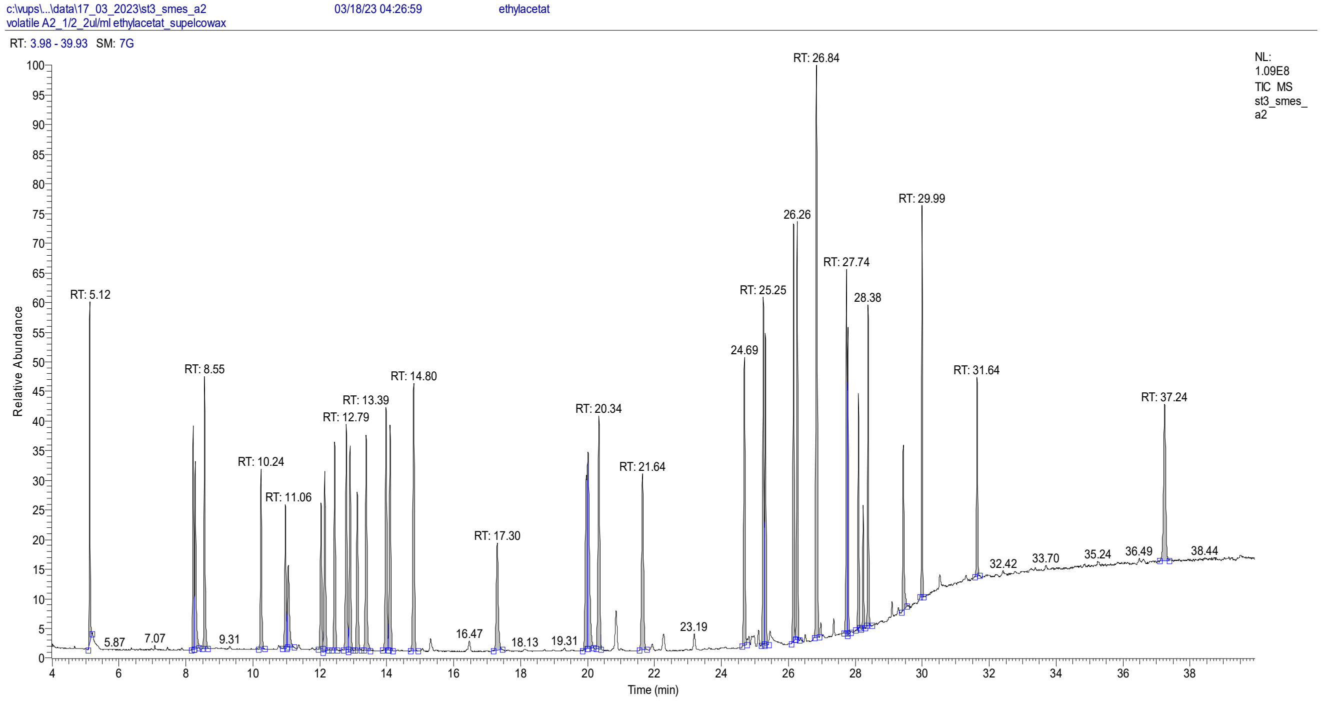

Figure S2. GC-MS chromatogram Allergen Mix 2.

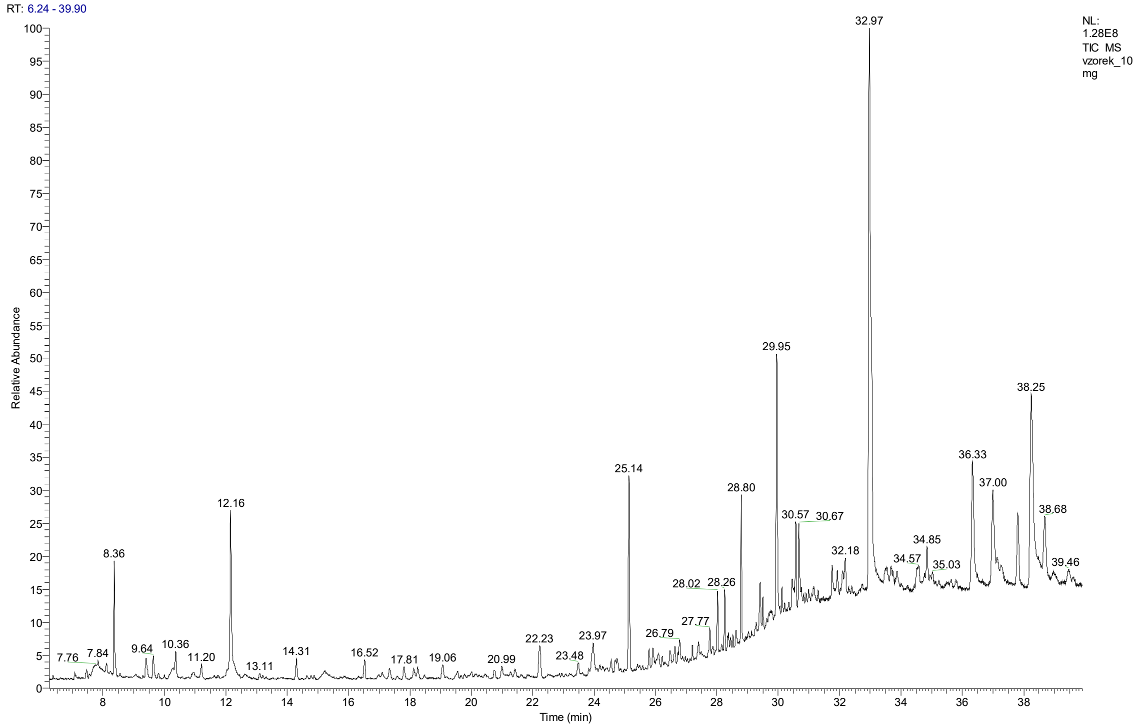

Figure S3. GC-MS chromatogram of *A.satureioides* lyophilized extract (10 mg).

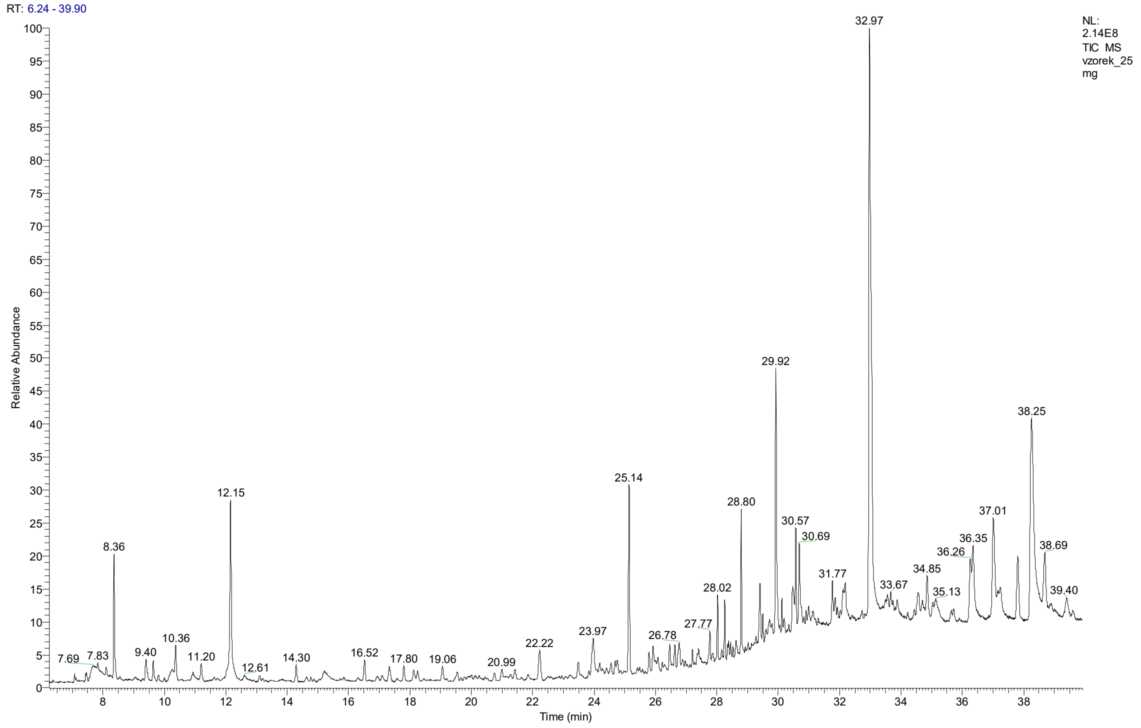

Figure S4. GC-MS chromatogram of *A.satureioides* lyophilized extract (25 mg).

Table S1. GC-MS Allergen mix 1 composition.

Retention time (RT)	Start RT	End RT	Substance	CAS number
3.29	3.24	3.33	beta-Pinene	18172-67-1
3.96	3.92	4.00	alpha-Terpinene	99-86-5
4.16	4.12	4.21	Limonene	5989-27-5
8.36	8.30	8.61	Linalool	78-70-6
9.39	9.33	9.58	beta-Caryophyllene	87-44-5
10.27	10.07	10.59	Menthol	89-78-1
11.20	11.10	11.32	alpha-Terpineol	98-55-5
13.15	12.97	13.33	Citronellol	106-22-9
14.30	14.15	14.40	trans-Anethole	4180-23-8
15.07	14.95	15.25	Geraniol	106-24-1
15.86	15.70	16.08	Benzyl alcohol	100-51-6
16.81	16.61	17.05	Ebanol 1+2	67801-20-1
23.15	23.00	23.29	Trimethyl-benzenepropanol	103694-68-4
23.49	23.42	23.62	Eugenol	97-53-0
25.97	25.90	26.00	Anise alcohol	105-13-5
26.03	26.00	26.13	Cinnamyl alcohol	104-54-1
26.75	26.69	26.93	Isoeugenol (E)	5932-68-1
27.03	26.96	27.05	trans,trans-Farnesol	106-28-5
27.08	27.05	27.15	alpha-Santalol	115-71-9
28.10	28.04	28.23	beta-Santalol	77-42-9
29.23	29.17	29.36	alpha-Amylcinnamyl alcohol	101-85-9
36.44	36.33	36.55	Sclareol	515-03-7

Table S2. GC-MS Allergen mix 2 composition.

Retention time (RT)	Start RT	End RT	Substance	CAS number
5.12	5.07	5.19	Terpinolene	586-62-9
8.22	8.17	8.25	Camphor	464-49-1
8.28	8.25	8.39	Benzaldehyde	100-52-7
8.55	8.48	8.66	Linalyl acetate	115-95-7
10.24	10.17	10.34	Methyl-2-octynoate	111-12-6
10.97	10.89	11.01	Citral - Neral	5392-40-5
11.06	11.03	11.22	Salicylaldehyde	90-02-8
12.04	11.95	12.08	Citral - Geranial	5392-40-5
12.15	12.09	12.25	Carvone	2244-16-8
12.44	12.36	12.52	Geranyl acetate	105-87-1
12.79	12.7	12.84	alpha-Damascone	43052-87-5
12.9	12.84	12.99	Dimethylbenzylcarbinyl acetate (DMBCA)	151-05-1
13.12	13.04	13.24	Methyl salicylate	119-36-8
13.39	13.31	13.51	beta-Damascone (E)	23726-91-2
13.98	13.88	14.03	delta-Damascone (Rose Ketone-3)	57378-68-4
14.1	14.05	14.18	beta-Damascenone (Rose Ketone-4)	23696-85-7
14.8	14.7	14.94	alpha-Isomethylionone	127-51-5
17.3	17.18	17.45	Hydroxycitronellal	107-75-5 2
19.97	19.85	19.98	Cinnamaldehyde	14371-10-9
20.02	20	20.18	Butylphenyl methylpropional	80-54-6
20.34	20.25	20.4	Tetramethylacetyloctahydro-naphthalene (ISO E®)	68155-67-9; 54464-57-2; 68155-66-8
21.64	21.55	21.78	Amyl salicylate	2050-08-0
24.69	24.62	24.76	alpha-Acetyl cedrene	32388-55-9
25.25	25.18	25.28	alpha-Amylcinnamaldehyde	78605-96-6
25.32	25.29	25.41	Eugenyl acetate	93-28-7
26.15	26.08	26.2	Galaxolide 1	1222-05-5
26.26	26.21	26.32	Galaxolide 2	1222-05-5
26.84	26.77	26.91	Hexadecanolactone / Dihydroambrettolide	109-29-5
27.74	27.65	27.76	Isoeugenyl acetate	93-29-8
27.78	27.76	27.86	3-Propylidene phthalide	17369-59-4
28.1	28.01	28.14	Coumarin	91-64-5
28.24	28.17	28.3	Hydroxyisohexyl 3-cyclohexene carboxaldehyde (HICC)	31906-04-4
28.38	28.33	28.5	Hydroxyisohexyl 3-cyclohexene carboxaldehyde (HICC)	31906-04-4
29.43	29.39	29.55	Vanillin	121-33-5
29.99	29.93	30.04	Benzyl benzoate	120-51-4
31.64	31.57	31.71	Benzyl salicylate	118-58-1
37.24	37.1	37.37	Benzyl cinnamate	103-41-3

Table S3. Fragrance allergen composition of *A.satureioides* lyophilized extract (10 mg).

Retention time (RT)	Start RT	End RT	Substance	CAS number
8.36	8.30	8.61	Linalool	78-70-6
9.40	9.33	9.58	beta-Caryophyllene	87-44-5
10.24	10.07	10.59	Menthol	89-78-1
11.20	11.10	11.32	alpha-Terpineol	98-55-5
12.16	12.09	12.25	Carvone	2244-16-8
14.31	14.15	14.40	trans-Anethole	4180-23-8
23.48	23.42	23.62	Eugenol	97-53-0
29.43	29.39	29.55	Vanillin	121-33-5

Table S4. Fragrance allergen composition of *A.satureioides* lyophilized extract (25 mg).

Retention time (RT)	Start RT	End RT	Substance	CAS number
8.36	8.30	8.61	Linalool	78-70-6
9.40	9.33	9.58	beta-Caryophyllene	87-44-5
10.24	10.07	10.59	Menthol	89-78-1
11.20	11.10	11.32	alpha-Terpineol	98-55-5
12.16	12.09	12.25	Carvone	2244-16-8
14.31	14.15	14.40	trans-Anethole	4180-23-8
23.49	23.42	23.62	Eugenol	97-53-0
29.43	29.39	29.55	Vanillin	121-33-5
